# The complete chloroplast genome sequence of *Spiraea japonica* var. *acuminata* Franch. (Rosaceae)

**DOI:** 10.1080/23802359.2022.2028590

**Published:** 2022-01-27

**Authors:** Qi Wang, Min-min Chen, Xia-fang Hu, Rui-hong Wang, Qiu-Ling He

**Affiliations:** Zhejiang Province Key Laboratory of Plant Secondary Metabolism and Regulation, College of Life Sciences and Medicine, Zhejiang Sci-Tech University, Hangzhou, China

**Keywords:** *Spirea japonica*; *Spirea japonica* var. *acuminata* Franch., chloroplast genome, phylogeny

## Abstract

*Spirea japonica* var. *acuminata* Franch. (Rosaceae) is a Chinese herbal medicine distributed in southwest and east China. The first complete chloroplast genome of *Spirea japonica* var. *acuminata* Franch. was assembled and reported in this study. The genome is 153,822 bp in length and contained 125 encoded genes in total, including 80 protein-coding genes, eight ribosomal RNA genes, and 37 transfer RNA genes. The phylogenomic analysis showed that *Spirea japonica* var. *acuminata* Franch. was closely related to *Spirea blumei, Spirea trilobata, Spirea mongolica* and *Spirea insularis* according to the current sampling extent.

*Spirea japonica* L. f. (1782) is a perennial shrubby species with pink flowers occurring in clusters at the tips of branches. It is widely dissimilar in 14 intraspecific varieties. *Spirea japonica* var. *acuminata* Franch. is one variant of *Spirea japonica* L. f. which is a Chinese herbal medicine classified in the Rosaceae. It is mainly distributed in southwest and east China (He et al. 2001; Zhang et al. 2006). The extracts from these plants were found to be bioactive for treating cough, anti-inflammation, headache, and analgesia (Li et al. 2002). In this study, we assembled and reported the first complete chloroplast genome of *Spirea japonica* var. *acuminata* Franch. The cumulative data will provide potential genetic resources for evolution of Rosaceae.

The leaves of *Spirea japonica* var. *acuminata* Franch. were collected from Hangzhou, Zhejiang, China (GPS: E120°14′14.95″, N30°23′58.95″). The specimen and extracted DNA was deposited at the College of Life Sciences and Medicine, Zhejiang Sci-Tech University (Zhejiang Province Key Laboratory of Plant Secondary Metabolism and Regulation, http://sky.zstu.edu.cn/, Qiu-Ling He and qlhe@zstu.edu.cn) under the voucher number ZSTULSM0001. The total genomic DNA was extracted from its fresh leaves using the CTAB method in accordance with the manufacturer’s instructions. The plastome sequences were generated using the Illumina HiSeq 2500 platform (Illumina Inc., San Diego, CA). In total, ca. 20.6 million high-quality clean reads (150 bp PE read length) were generated with adaptors trimmed. These clean data were *de novo* assembled to complete chloroplast genome using GetOrganelle (Jin et al. 2020).The assembled cp genome was annotated using Geneious v11.1.5 (Drummond 2012) with *Spirea trilobata* plastome (GenBank: MW822176) as a reference.

The full length of *Spirea japonica* var. *acuminata* Franch. chloroplast sequence (GenBank Accession No. MZ981784) is 153,822 bp, consisting of a large single copy region (LSC with 82,227 bp), a small single copy region (SSC with 18,907 bp), and two inverted repeat regions (IR with 26,344 bp). The overall GC content of *Spirea japonica* var. *acuminata* Franch. chloroplast genome was 36.6% and the GC content of the LSC, SSC, and IR regions are 34.3%, 30.3%, and 42.5%. A total of 125 genes were contained in the genome (80 protein-coding genes, eight rRNA genes, and 37 tRNA genes). Sixteen genes had two copies, which were comprised of fine PCG genes (*ndhB, rps7, ycf2, rpl2, rpl23*), seven tRNA genes (*trnI-CAU, trnV-GAC, trnI-GAU, trnA-UGC, trnR-ACG, trnN-GUU, trnL-CAA*), and all four rRNA species (*rrn16, rrn23, rrn4.5, rrn5*). In the genome, nine protein-coding genes (*atpF, rpl2, ndhB, rps16, rpoC1, clpP, rpl16, petD, petB*) had one intron, *rps12* and *ycf3* gene contained two introns.

To confirm the phylogenetic position of *Spirea japonica* var. *acuminata* Franch., we obtained 11 published complete chloroplast genomes of Rosaceae from NCBI. *Hippophae neurocarpa* was used as outgroup for constructing the phylogenetic tree. The 13 complete cp sequences were aligned using MAFFT v7.3 (Katoh and Standley 2013). The maximum-likelihood (ML) phylogenetic analyses were constructed using IQTREE v1.6.7 (Nguyen et al. 2015) with 5000 bootstraps under the TVM + F+R3 substitution model. The phylogenetic tree revealed that *Spirea japonica* var. *acuminata* Franch. was closely related to *Spirea blumei, Spirea trilobata, Spirea mongolica* and *Spirea insularis* according to the current sampling extent (Figure 1).

**Figure 1.**
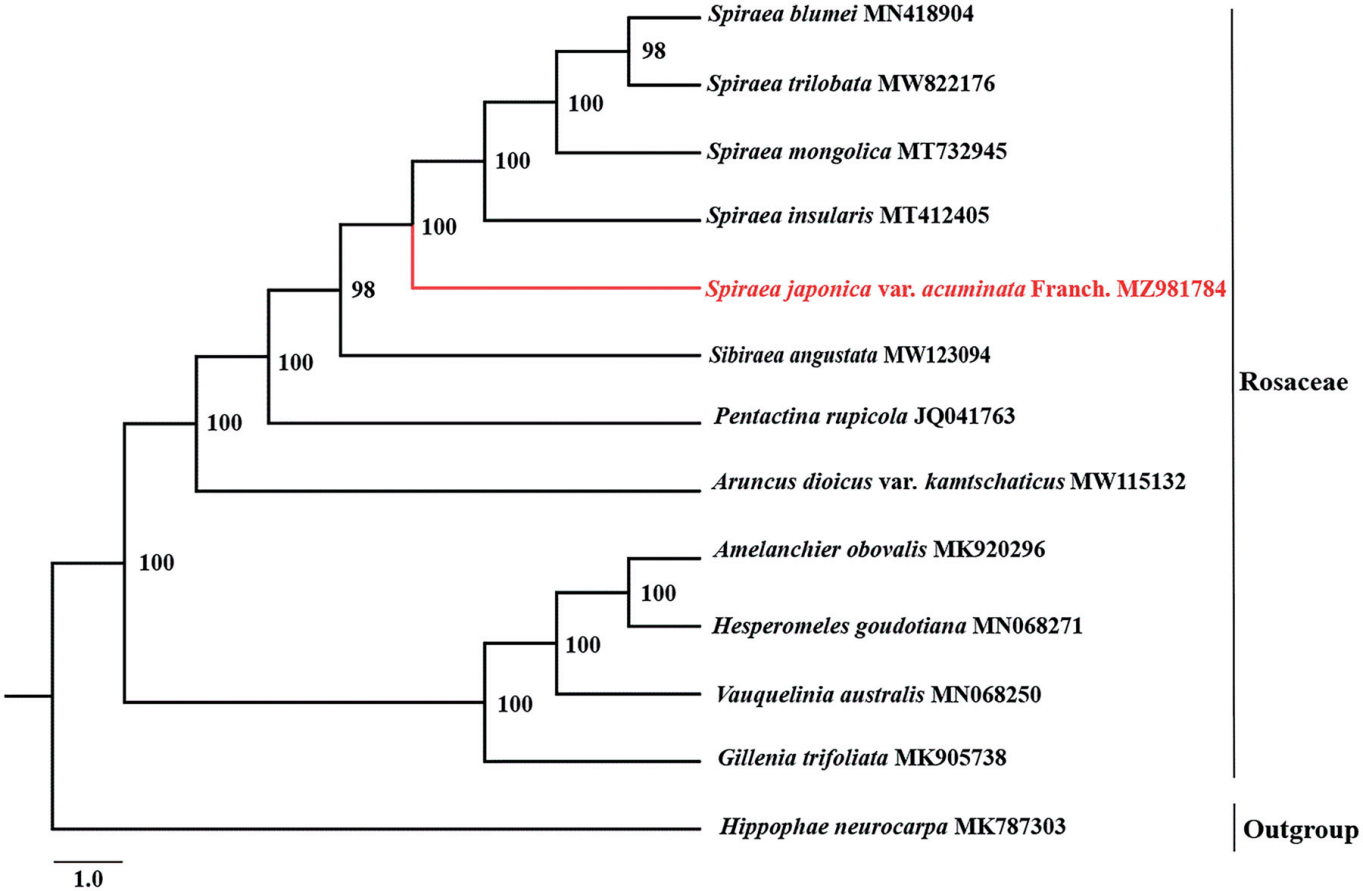
Phylogenetic relationship of *Spirea japonica* var. *acuminata* Franch. in Rosaceae using maximum likelihood (ML) method based on 12 species complete chloroplast genomes (accession numbers were listed behind each taxon. Statistical support values were showed on nodes.).

## Data Availability

The genome sequence data that support the findings of this study are openly available in GenBank of NCBI (https://www.ncbi.nlm.nih.gov) under the Accession no. MZ981784. The associated BioProject, SRA, and Bio-Sample numbers are PRJNA759429, SRR15685682, and SAMN21168751, respectively.
